# Hyalinizing spindle cell tumor with giant rosettes

**DOI:** 10.4103/0256-4947.70575

**Published:** 2011

**Authors:** Anikode S. Ramaswamy, K. Ramakantha Chatura

**Affiliations:** From the Department of Pathology, People’s Education Society Institute of Medical Sciences and Research, Kuppam, Jagadguru Jayadeva Murugarajendra Medical College, Davangere, India

## Abstract

Low-grade fibromyxoid sarcomas are uncommon deep-seated soft tissue neoplasms that exhibit a deceptively benign appearance microscopically. The finding of a linear or whorled array of spindled cells with few or no mitoses in a characteristic myxoid stroma can pose diagnostic dilemmas. Recurrences are common, and late metastases have been recorded. A closely related tumor, the so-called hyalinizing spindle cell tumor with giant collagen rosettes (HSCTGR), has also been described, with both the neoplasms having a similar cytogenetic abnormality and clinical behavior. Because of the similarities, both lesions are considered to be a single entity within the spectrum of low-grade sarcomas. Two cases of HSCTGR occuring in the lower limb are described in this report.

There have been major conceptual changes in the understanding of the fibroblastic/myofibroblastic tumors as can be seen from the revised WHO classification of these lesions. Low-grade fibromyxoid sarcoma (LGFMS) is one recently recognized entity. This lesion is unique in that most of the time it goes unrecognized because of its bland morphology. Consequently, fewer than 150 cases of this lesion have been reported worldwide. The hyalinizing spindle cell tumor with giant rosettes (HSCTGR), which is now accepted as a subtype of LGFMS, is much more rarely described.^1^ Two such cases that were encountered over a 4-year period are reported here.

## CASES

The first case was a 30-year-old female who presented with a slow-growing painless swelling over the back of the thigh that had been present for 5 years. There was no regional lymphadenopathy or any other contributory findings in the clinical history. The second case, a 25-year-old male, also presented with a similar swelling that had been present for 5 years. Intraoperatively, the masses were well circumscribed; it was partly solid and partly cystic in the first case, and solid and adherent to the aponeurosis of the hamstring muscles in the second case. Provisional clinical differential diagnoses of neurofibroma and fibrosarcoma were made, and the excised masses were sent for histopathological examination.

**Figure 1:**
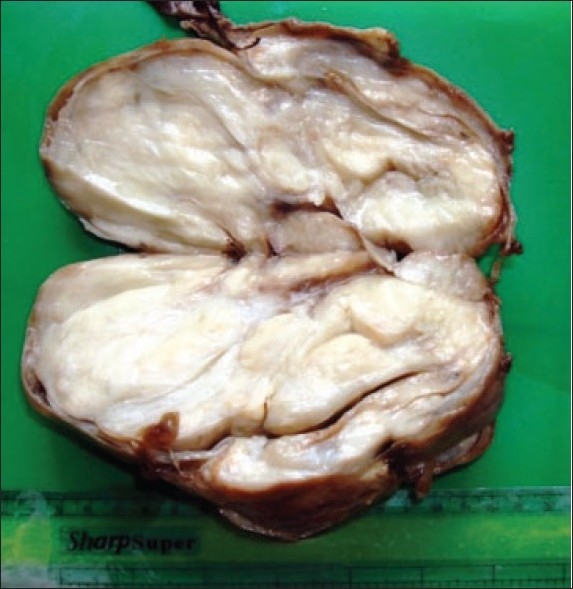
Solid and gray-white mass with myxoid areas.

In the first case, the mass was globular and circumscribed, measuring 18×18×9 cm in gross appearance. On cut section, a central cystic area was seen that was filled with grayish-white friable tissue; this central area was bordered by a peripheral solid area. In the second case, the lesion measured 15×10×8 cm and was solid and gray-white, and had myxoid areas (**Figure 1**).

**Figure 2:**
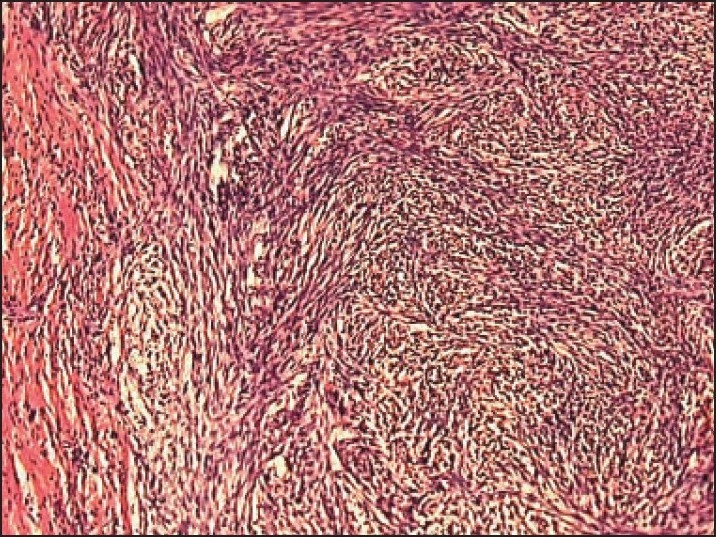
Spindle-shaped cells having a fascicular and vaguely storiform pattern (hematoxylin and eosin, ×10).

**Figure 3:**
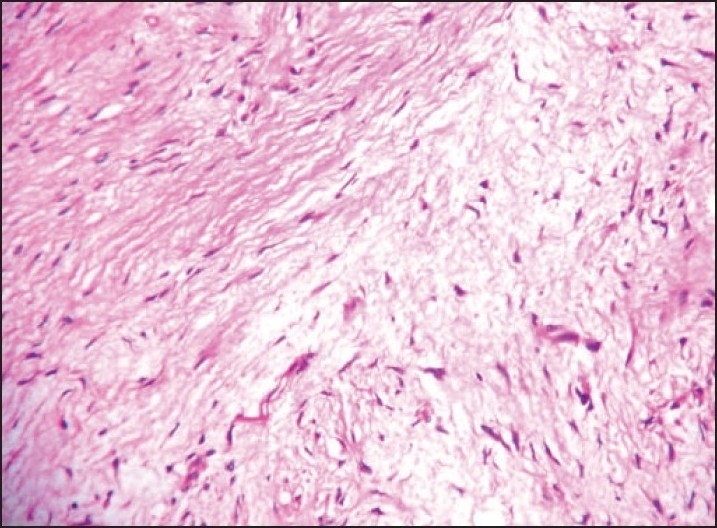
Exceedingly myxoid stroma (hematoxylin and eosin, ×40).

**Figure 4:**
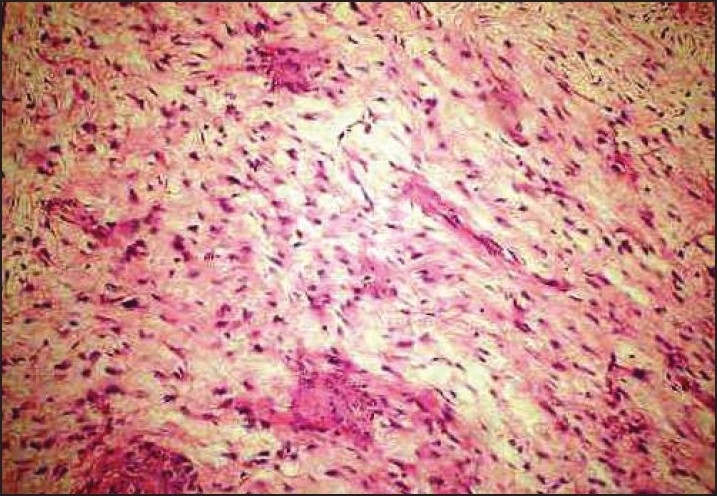
Myxoid zones composed of bland spindled cells with delicate branching-to-curvilinear vessels (hematoxylin and eosin, ×10).

Microscopic examination in both cases revealed well-circumscribed tumors composed of spindle-shaped, bland-looking cells having a fascicular and a vaguely storiform pattern (**Figure 2**). The cellularity varied. Some areas showed spindle cells arranged in irregular crisscrossing fascicles separated by a moderate amount of collagen, often with a ‘cracking’ artifact around the elongated fibroblastic cells. In other areas there was extensive stromal hyalinization, with a paucity of neoplastic cells, and in yet others, the stroma was exceedingly myxoid (**Figure 3**), with widespread separation of the neoplastic cells. In the myxoid zones, the tumor was composed of bland spindled-to-stellate cells in a myxoid-to-lightly collagenized stromal background, often with associated curvilinear-to-branching prominent vasculature (**Figure 4**). At places the spindle cells had a wavy irregular outline. There was no mitotic activity detectable.

**Figure 5:**
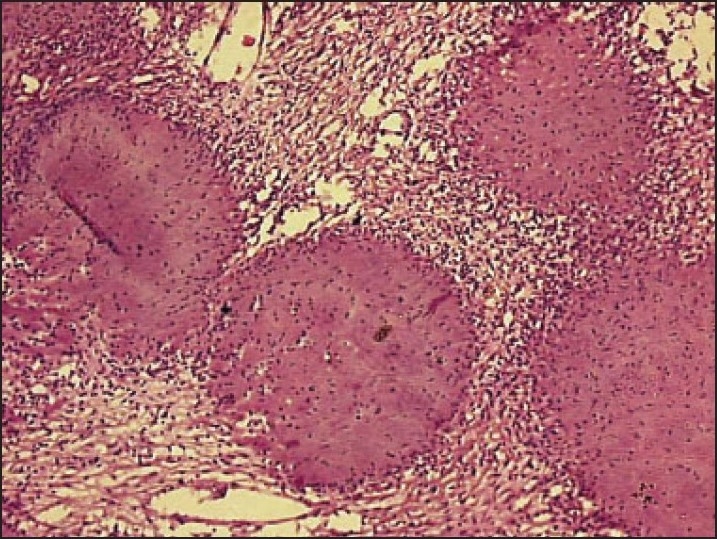
Spindled stroma punctuated by large collagen rosettes (hematoxylin and eosin, ×10).

The spindled stroma was punctuated by large collagen rosettes ringed by nuclei (**Figure 5**). They were randomly distributed, primarily in the cellular component, and were ovoid and individualized or clustered, or assumed the form of large irregularly shaped hyalinized bodies. The center of these rosettes was composed of hyalinized collagen as demonstrated by the Masson trichrome stain. No cell processes were evident in the hyalinized area. These were surrounded by rounded-to-ovoid cells that had clear-to-eosinophilic cytoplasm, with no nuclear atypia or mitotic activity. These cells were distinctly different from the surrounding spindle-shaped cells. This cellular cuff around the rosettes varied from a few to several cells thick. The nuclei were sometimes stacked in bent columns arranged perpendicular to the edge of the hyalinized body. At places these rosettes coalesced into long serpiginous cords or broad zones of dense hyalinization. Some hyalinized bodies with scant peripheral nuclei formed round hyalinized mats.

**Figure 6:**
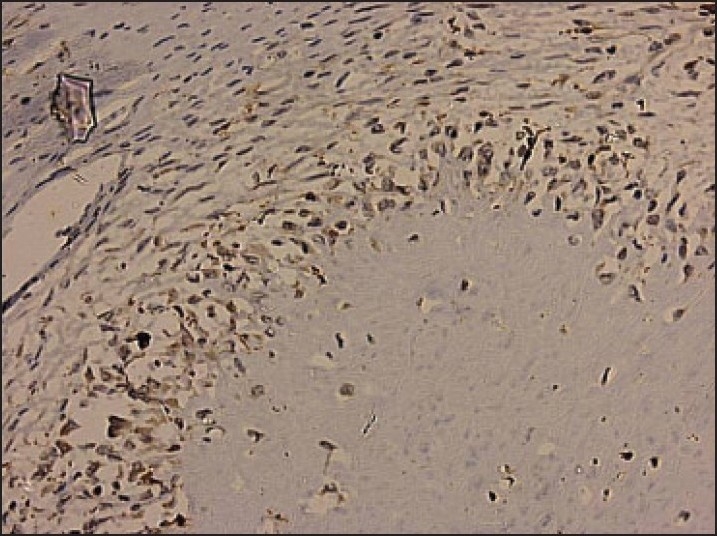
S-100 positivity in the rounded cells present in the cellular cuff of the giant rosettes (hematoxylin and eosin, ×40).

Immunohistochemically, both the spindle-shaped and ovoid cells stained strongly for vimentin. S-100 was frequently positive in the rounded cells (**Figure 6**). The spindle-shaped cells stained negative for S-100. Negativity for S-100 militates against the diagnosis of schwannoma and strongly favors a diagnosis of HSCTGR. The above conglomeration of histomorphological and immunohistochemical features led us to the diagnosis of HSCTGR.

## DISCUSSION

Soft tissue tumors composed of malignant fibroblastic cells and their variants, once thought to be rare, are now believed to represent a large group of soft tissue sarcomas. The category of fibrosarcoma has been expanded to include a number of distinct subtypes.^1^,^2^ Evans in 1987 described a distinctive form of fibrosarcoma that he called ‘low-grade fibromyxoid sarcoma’ (LGFMS).^3^,^4^ A decade after these seminal studies, Lane et al described the entity called ‘hyalinizing spindle cell tumor with giant rosettes’ (HSCTGR).^2^ These two entities share many histologic features and it has been proposed that these tumors represent a morphologic spectrum of the same entity.^1^–^3^

LGFMS is a distinctive variant of fibrosarcoma characterized by an admixture of heavily collagenized and myxoid areas, deceptively bland spindle cells arranged in a whorling pattern, and arcades of curvilinear blood vessels.^1^,^5^ These are rare sarcomas which are commonly seen in young to middle-aged adults and typically occur in a subfascial location in the proximal extremities or trunk as painless, deep-seated, slowly-enlarging soft tissue masses.^1^,^4^,^5^ Grossly, the tumor is an oval multilobulated mass, ranging in size from 2 to 20 cm in diameter. Although most of the lesions appear well circumscribed, they can extensively infiltrate the surrounding soft tissue.^3^,^5^ The cut surface has a whorled white-tan appearance. Cystic degeneration, which was prominent in our first case, is an uncommon finding.^5^ Our cases did not show areas with increased cellularity and nuclear atypia as is seen in the usual type of fibrosarcomas of intermediate grade.^6^,^7^

In approximately 40% of cases of LGFMS the stroma is punctuated by the presence of a variable number of large rosette-like structures that merge imperceptibly with the surrounding hyalinized or spindled stroma. Both our cases showed these rosettes. The term HSCTGR has been used for those LGFMS where the collagen rosettes are particularly prominent and well-formed.^1^–^3^,^5^,^7^,^8^

The immunohistochemical profile, especially in HSCTGR, has suggested a neural phenotype, with the cells forming the rosettes being Leu-7, S-100, and pgp 9.5 positive and the spindle cells usually being negative.^2^,^3^,^5^ An important cytogenetic tool which will be helpful in the diagnosis of this lesion has been the recognition of a characteristic balanced translocation t(7; 16) (q34:p11) and the finding of a novel fusion gene FUS/CREB3L2.^3^

The microscopic differential diagnoses of LGFMS include low-grade myxofibrosarcoma, myxoid neurofibroma, and myxoid solitary fibrous tumor. Low-grade myxofibrosarcoma has more cellular atypia and less swirling of tumor cells in a uniformly myxoid stroma. Myxoid neurofibroma shows more wavy nuclei of the spindle cells and strong S-100 positivity. The osteoid, in rare cases of osteosarcoma, may be confused for the collagen rosettes. Calcification in the material and the surrounding marked nuclear pleomorphism will help in settling the issue.^2^–^6^ Though the lesion appears bland in morphology, long-term follow-up has revealed that it has a potential for metastasis, especially to the lungs.^1^,^3^,^8^ Improved recognition and treatment have improved the prognosis of the lesion but, nevertheless, prolonged follow-up is necessary.
